# Ultrasound evolution of parenchymal changes in the thyroid gland with autoimmune thyroiditis in children prior to the development of papillary thyroid carcinoma – a follow-up study

**DOI:** 10.3389/fendo.2023.1172823

**Published:** 2023-04-12

**Authors:** Dominika Januś, Monika Kujdowicz, Małgorzata Wójcik, Anna Taczanowska-Niemczuk, Aleksandra Kiszka-Wiłkojć, Wojciech Górecki, Jerzy B. Starzyk

**Affiliations:** ^1^ Department of Pediatric and Adolescent Endocrinology, Chair of Pediatrics, Institute of Pediatrics, Jagiellonian University Medical College, Krakow, Poland; ^2^ Department of Pediatric and Adolescent Endocrinology, University Children Hospital in Krakow, Krakow, Poland; ^3^ Department of Pathomorphology, Jagiellonian University Medical College, Krakow, Poland; ^4^ Department of Pathology, University Children Hospital in Krakow, Krakow, Poland; ^5^ Department of Pediatric Surgery, Institute of Pediatrics, Jagiellonian University Medical College, Krakow, Poland; ^6^ Department of Pediatric Surgery, University Children Hospital in Krakow, Krakow, Poland

**Keywords:** autoimmune thyroiditis, papillary thyroid carcinoma, ultrasonography of thyroid gland, TgAb/TPOAb ratio, hypoechogenic thyroid nodules

## Abstract

**Background:**

Follicular cell-derived thyroid carcinoma represents the vast majority of paediatric thyroid cancers (TCs). Papillary thyroid carcinoma (PTC) accounts for over 90% of all childhood TC cases, and its incidence in paediatric patients is increasing. The objective of this follow-up study was to present the outcome of ultrasound (US) and laboratory monitoring of paediatric patients with autoimmune thyroiditis (AIT) prior to the development of PTC.

**Patients and methods:**

This prospective study included 180 children and adolescents (132 females; 73.3%) with a suspicion of thyroid disorder referred to the Outpatient Endocrine Department. The patients were divided into four groups: 1) 28 patients with a mean age of 10.7 [standard deviation (SD), 3.1] y, in whom PTC was detected during the active surveillance of AIT [AIT(+), PTC(+) follow up (F)]; 2) 18 patients with a mean age of 12.8 (SD, 3.4) y, in whom PTC and AIT were detected upon admission (A) [AIT(+), PTC(+) A]; 3) 45 patients with a mean age of 13.0 (SD, 3.4) y, in whom PTC was detected upon admission and AIT was excluded [AIT(-), PTC(+) A]; and 4) an age- and sex-matched control group of 89 patients with AIT and with a mean age of 9.4 (SD, 3.0) y. The analysis included clinical, US, and laboratory assessment results of children on admission (groups 1–4) and during follow-up (groups 1 and 4) in the Paediatric Endocrine Outpatient Department.

**Results:**

Upon admission of those in group 1, the US evaluation revealed a hypoechogenic thyroid gland in 12 and an irregular normoechogenic gland in 16 patients. US monitoring revealed an increase in thyroid echogenicity and an increased irregularity of the thyroid structure during the follow-up period of all of the patients from group 1. Such changes were not noticed in group 4. PTC was diagnosed at the mean time of 3.6 y (3 mo–9 y) since AIT confirmation in group 1. The mean maximum PTC diameter as per the US was significantly smaller in group 1 than in groups 2 and 3 [13.2 (10.8) mm vs. 22.2 (12.8) and 22.05 (15.4) mm]. Fewer patients in group 1 were referred to 131I than in groups 2 and 3 (71.4% vs. 94.4 and 93.3%). Interestingly, significant differences were observed in the thyroglobulin antibody (TgAb)/thyroid peroxidase antibody (TPOAb) ratio between groups 2 and 3, as opposed to group 4, at the beginning of observation [15.3 (27.6) and 3.5 (8.8] vs. 0.77 (1.9)]. In group 1, after the follow-up, an increase in the TgAb/TPOAb ratio was observed [1.2 (9.8) to 5.2 (13.5)]. There were no significant differences between groups 1–3 in labeling index Ki67, lymph nodes metastasis, extrathyroidal extension, and angioinvasion. There were no associations between thyroid-stimulating hormone, TgAb, and the extent of the disease.

**Conclusion:**

The use of thyroid US focused on the search for developing tumours in the routine follow-up of patients with AIT may not only help in the early detection of thyroid malignancies that are not clinically apparent but may also influence the invasiveness of oncological therapy and reduce the future side effects of 131I therapy. We propose that the repeat evaluation of TPOAb and TgAb warrants further exploration as a strategy to determine TC susceptibility in paediatric patients with AIT in larger multicentre studies.

## 1 Introduction

Follicular cell-derived thyroid carcinoma represents the vast majority of paediatric thyroid cancers (TCs) (1). Papillary thyroid carcinoma (PTC) accounts for over 90% of all childhood TC cases (2). The most recent statistical data presented by Siegel et al. estimate that TC accounts for 12% of cancers in adolescents and 2%, in children below 14 y of age (3). TC ranks the fourth most common type of cancer in adolescents and the seventh most common in children in the United States (3). According to the data of the Polish National Cancer Registry, new cases of TC in patients below 19 y of age constitute 2.3% of all TCs diagnosed, every second solid neoplasm in girls, and every eighth solid neoplasm in boys (2).

The prevalence of chronic autoimmune thyroiditis (AIT) has been assessed as up to 2% in children and almost 10% in adolescents, depending on the populations studied (4, 5). The disease has female sex predominance, and although it can be diagnosed from infancy, its peak is observed after puberty (6). AIT is responsible for approximately 55–65% of all pediatric euthyroid goiters (7, 8). In 1955, Dailey et al. suggested for the first time that AIT might be considered a premalignant lesion, as chronic inflammation contributes to the development of cancer in many tissues (9–12). Chronic autoimmune inflammation of the thyroid may cause functional and/or structural thyroid disorders leading to an estimated one-third of patients to develop a nodular rebuilding of the thyroid gland (13–20).

In the paediatric population, the incidence of thyroid nodules is lower (0.5–2%) than in those with AIT (3.5–31.5%) (13–23). In recent years, there has been an increase in the coincidental occurrence of AIT and PTC in children and adolescents (7, 14, 16, 23). According to the current paediatric guidelines, neck US in children with autoimmune thyroid disease (AITD) should be performed at least annually (2, 24).The practicality of this approach was presented by our group in the study evaluating parenchymal changes in the thyroid gland with diffuse AIT in children prior to the development of PTC (25). In 2022, Siegel et al. in Cancer Statistics revealed that in the adult population in the United States, after decades of increase, TC incidence rates have now been declining partly because of recent changes in clinical practice designed to reduce overdetection. However, in a more recent analysis in 2023, Huang et al. found that the incidence rate of TC increased in most countries among individuals irrespective of age groups (3, 26). Additionally, the incidence rate increased in populations aged <40 y in several countries, including Poland (26). Because the presentation of PTC in children is more severe than in adults, the extent of surgery is larger, and therapy also includes 131I therapy, leading to potential future therapeutic side effects. On the other hand, overall survival in children is excellent (24, 27, 28). Therefore, it is important to focus the research on the early detection of malignancies with the aim to reduce future side effects of oncological therapy for PTC in children with long life-expectancy (24, 27, 28).

The present study provides an expansion of previous work from our centre related to the monitoring of patients with thyroid disorders (25). The aim of this study was to prospectively present the outcome of US follow-up in children with AITD who developed PTC and to characterize the US and laboratory variables prior to the development of PTC that might be useful in selecting the PTC risk group within the large population of paediatric patients with AIT.

## 2 Patients and methods

This prospective study included 180 children and adolescents (132 females; 73.3%) referred to the Outpatient Endocrine Department. The patients were recruited since 2010 till 2023. Patients were divided into four groups: 1) 28 patients with a mean age of 10.7 [standard deviation (SD), 3.1] y, in whom PTC was detected during the active surveillance of AIT [AIT(+), PTC(+) follow up (F)]; 2) 18 patients with a mean age of 12.8 (SD, 3.4) y, in whom PTC and AIT were detected upon admission (A) [AIT(+), PTC(+) A]; 3) 45 patients with a mean age of 13.0 (SD, 3.4) y, in whom PTC was detected upon admission and AIT was excluded [AIT (–), PTC(+) A]; and 4) an age- and sex-matched control group of 89 patients with AIT and with a mean age of 9.4 (SD, 3.0) y.

The prospective analysis of medical records included the evaluations of thyroid function status, US, and cytological and histopathological variables in patients with PTC. All hormonal and immune assessments were routinely performed at the Department of Biochemistry at the University Children’s Hospital in Krakow, Poland and were determined using a single fasting blood sample. Thyroid-stimulating hormone (TSH), fT3, and fT4 levels were measured using immunochemistry with an ADVIA Centaur machine, and thyroid peroxidase antibody (TPOAb), thyroglobulin antibody (TgAb), and thyroid hormone receptor antibody (TRAb) levels were assessed using the radioimmunoassay method with a Brams machine. TSH, fT3, fT4, TPOAb, and TgAb assessments were performed upon admission to the Department (on first visit) and prior to therapy with levothyroxine or antithyroid methimazole when it was needed. TPOAb and TgAb were additionally examined if US analysis revealed a nodule (prior to surgery). TRAb was controlled only in patients with thyrotoxicosis. TSH, fT3, and fT4 were controlled routinely every 3, 4, and 6 mo in all of the patients. Either TPOAb or TgAb were assessed in all patients. Most of the patients underwent all of the assessments of TPOAb and TgAb. In group 1, TPOAb and TgAb were both assessed in 23 of the 28 patients. In group 2, TPOAb was assessed in all 18 and TgAb, in 10 of the 18 patients. In group 3, TPOAb was assessed in 33 and TgAb, in 25 of the 45 patients. In group 4, TPOAb was assessed in 85 and TgAb, in 69 of the 89 patients. Therefore the TgAb/TPOAb ratio could only be assessed in all 23 patients in group 1, 10 out of 18 patients in group 2, 23 out of 45 patients in group 3, and 66 out of 89 patients in group 4.

AIT was diagnosed based on the typical features of chronic AIT seen during thyroid US assessment as previously described, on increased TPOAb and/or TgAb and/or TRAb antibodies levels, and after histopathological confirmation in all of the patients after thyroidectomy (groups 1 and 2) (16, 25). In group 4 the diagnosis of AIT was based on positive TPOAb and/or TgAb and ultrasound.

Thyroid US was performed on all of the patients at the time of thyroid dysfunction diagnosis and annually since then. US of the thyroid gland was performed at the University Children’s Hospital by paediatric endocrinologists and surgeons with experience in paediatric US (DJ and AT >20 y and AKW and MW >15 y). Thyroid US was performed using a high-resolution Voluson 730 GE Medical System (8- to 12-MHz linear-array transducer), Philips Epiq5 (L12-5 linear transducer), Philips iE22 (L11-3 linear transducer), and Samsung HS40 (LA3-16AD transducer). The US examination was performed in the longitudinal and transversal planes. Normal thyroid parenchyma (normoechogenic background) was defined as demonstrating homogenous echogenicity and relative hyperechogenicity compared with the adjacent sternohyoid, sternothyroid, omohyoid, and sternocleidomastoid muscles as described previously (16, 25). The analysis included US features of the thyroid gland according to the EU-TIRADS PL 2022 classification (Polish update of EU-TIRADS 2017) and of the lymph nodes (29, 30).

The patients presenting a nodule containing suspicious features, such as hypoechogenicity, a hyperechogenic ‘border’ between a nodule and thyroid parenchyma, poorly defined margins, irregular shape, microcalcifications, solid composition, presence of chaotic vascularity as detected *via* Doppler flow, and/or pathological lymph nodes were referred to paediatric oncologic surgeons for fine needle aspiration biopsy (FNAB).

FNAB results were classified according to Bethesda criteria (31). In the patients with PTC, total thyroidectomy with a histopathological verification of the lateral and central lymph nodes was performed. Histopathological evaluation was performed in the Department of Pathology of University Children’s Hospital in Krakow.

The hematoxylin and eosin (HE)-stained tissue slides (deparafinnated, cut with 3.5-um thickness) were scanned with the NanoZoomer SQ Hamamatsu (x400 magnification) after a routine diagnosis of thyroid nodules. The pictures were taken from the scans, with the scale placed on the bottom left.

This study was approved by the relevant institutional review board (The Bioethics Committee of the Jagiellonian University; opinion number:1072.6120.288.2021). Written informed consent was obtained from all of the participants and/or their parents. Written informed consent was obtained from the individual(s) and minor(s)’ legal guardian/next of kin for the publication of any potentially identifiable images or data included in this article.

### 2.1 Statistics

We summarised baseline and demographic characteristics using descriptive statistics. Categorical variables were expressed as percentages. We assessed the distribution of continuous variables using the Shapiro-Wilks test and described continuous variables using mean and SD when appropriate. We used the non-parametric Kruskal-Wallis and U-Mann Whitney tests to compare the groups of patients. For the assessment of correlations, we used the Spearmann test. A two-tailed p<0.05 was considered statistically significant. All of the analysis were performed with STATISTICA, version 13 (TIBCO Software Inc., Palo Alto, CA 94304 United States).

## 3 Results

Clinical, hormonal and histopathological assessment of the patients in the four groups are presented in **Table 1** and **Table 2**.

**Table 1:** AIT-confirmation –first visit.

parameters	AIT confirmation-first visit			AIT control group	p value
	(1) AIT(+) PTC(+) F	(2) AIT(+) PTC(+) A	(3) AIT (–) PTC(+) A	(4) AIT(+) PTC(-)	
n	28	18	45	89	
female n [%]	23 [82.1]	14 [77.8]	31 [68.9]	64 [71.9]	p=0.1
Family history of thyroid diseases n [%]	21 [75]	7 [38.9]	6 [13.3]*	47 [52.8]*	p=0.047
Cause of referral n [%] USG Goiter	22 [78.6] 2 [21.4]	7 [38.9] 11 [61.1]	17 [37.8] 28 [62.2]	9 [10.1] 80 [89.9]	p=0.1 p=0.2
Age at AIT diagnosis (years) mean [SD]	10.7 [3.1]	12.8 [3.4]	13.0 [3.4]	9.4 [3.0]	p=0.1
Thyroid volume (ml) mean [SD]	18.4 [17.0]	25.1 [15.3]	27.2 [18.7]	19.9 [18.1]	p=0.2
TSH at AIT diagnosis (µIU/ml N:0.4-4.0) mean [SD]	8.6 [4.9]	4.8 [7.9]	2.5 [1.3]*	44.3 [115.6]*	p=0.02
TPOAb at AIT diagnosis (IU/ml N<30) mean [SD]	2134.9 [3780.7]*	888.8 [2093.9]*	19 [13.5]*	4628.7 [3533.2]*	p=0.01
TgAb at AIT diagnosis (U/ml N<30) mean [SD]	449.8 [476.9]	1059.8 [2467.5]	14.47 [8.5]*	1176.3 [2374.8]*	p=0.01
TgAb/TPOAb mean [SD]	1.2 [9.8]	15.3 [27.6]	3.5 [8.8]*	0.77 [1.9]*	p=0.04

Clinical, hormonal and histopathological assessment of patients in four groups: 1- PTC detected during active surveillance of AIT [AIT(+) PTC(+) F], 2- PTC and AIT detected on admission [AIT(+) PTC(+) A], 3- PTC detected on admission, AIT excluded [AIT (–) PTC(+) A], 4- Age-, and gender matched control group of pediatric patients with AIT. Legend: AIT-autoimmune thyroiditis, PTC-papillary thyroid carcinoma, significant differences between the groups are marked with asterix for p<0.05. Significantly higher TPOAb levels were found in group 4 than in other groups.

**Table 2:** PTC diagnosis.

parameters	PTC follow-up	PTC on admission		AIT control group	p values
	(1) AIT(+) PTC(+) F	(2) AIT(+) PTC(+) A	(3) AIT(-) PTC(+) A	(4) AIT(+) PTC(-)	
n	28	18	45	89	
Time to PTC detection since referral in group 1 and time of follow-up in group 4 (years) mean [range]	3.6 [0.3-9]	–	–	4.3 [0.2-12.6]	p=0.4
Age at PTC diagnosis (years) mean [SD]	14.5 [3.3]	12.8 [3.4]	13.0 [3.4]	13.7 [3.1]	p=0.4
Thyroid volume at PTC diagnosis (ml) mean [SD]	17.05 [14.9]	25.1 [15.3]	27.2 [18.7]	14.5 [5.8]	p=0.4
PTC largest diameter (mm) mean [SD]	13.2 [10.8]*	22.2 [12.8]*	22.05 [15.4]*	–	p=0.04
unifocal n[%]	13 [46.4]	4 [22.2]	15 [33.3]	–	p=0.3
multifocal & bilateral n[%]	15 [53.6]	14 [77.8]	30 [66.7]	–	p=0.2
LNM largest diameter (mm) mean [SD]	4.6 [7.5]	7.9 [8.8]	5.03 [7.8]	–	p=0.2
LNM central n [SD]	5.0 [11.3]	6.3 [9.8]	3.2[5.1]	–	p=0.3
LNM lateral n [SD]	2.0 [4.9]	4.9 [6.1]	2.4 [4.9]	–	p=0.4
LI Ki67 mean [SD]	9.6 [4.6]	10.15 [4.9]	9.7 [5.2]	–	p=0.4.
ETE n[%]	10 [35.7]	6 [33.3]	21 [46.6]	–	p=0.4
AI n[%]	11 [39.3]	8 [44.4]	25 [55.5]	–	p=0.3
TSH at PTC dgn (µIU/ml N:0.4-4.0) mean [SD]	3.9 [5.2]	4.8 [7.9]	2.5 [1.3]	2.7 [3.9]	p=0.4
TPOAb at PTC dgn (IU/ml N<30) mean [SD]	957.9 [2025.3]*	888.8 [2093.9]*	19 [13.5]*	3966.1 [2978.2]*	p=0.01
TgAb at PTC dgn (U/ml N<30) mean [SD]	758.9 [1567.0]	1059.8 [2467.5]	14.47 [8.5]*	853 [1265.8]*	p=0.01
TgAb/TPOAb mean [SD]	5.2 [13.5]	15.3 [27.6]	3.5 [8.8]*	0.21 [0.4]*	p=0.04
^131^I n [%]	20 [71.4]	17 [94.4]	42 [93.3]	–	p=0.4
PTC subtypes n classic follicular c,f c,f,s cs columnar ds fs c,f,s,c/m c,f,s,t,c/m ds,s,f c,f,s,o,g s,anaplastic	14 4 4 4 1 1 - - - - - - -	7 3 - 3 2 - 1 1 1 - - - -	24 8 4 3 - - 1 1 - 1 1 1 1	–	

Clinical, hormonal and histopathological assessment of patients in four groups: 1- PTC detected during active surveillance of AIT [AIT(+) PTC(+) F], 2- PTC and AIT detected on admission [AIT(+) PTC(+) A], 3- PTC detected on admission, AIT excluded [AIT(-) PTC(+) A], 4- Age-, and gender matched control group of pediatric patients with AIT. Legend: AIT-autoimmune thyroiditis, PTC-papillary thyroid carcinoma, LNM-lymph node metastasis, LN-lymph nodes, C LNM- central lymph nodes metastasis, L LNM-lateral lymph nodes metastasis, ETE-extrathyroidal extension, AI- angioinvasion, PTC subtypes: c-classic, f-follicular, s-solid, ds-diffuse sclerosing, c/m-cribriform morular, t-tall cell, g-glomerular, o-oncocytic; F-follow up, A-admission, AI-angioinvasion, ETE-extrathyroidal extension; significant differences between the groups are marked with asterix for p<0.05. Significantly larger PTC diameters were found in groups 2 and 3 than in group 1. Significantly higher TPOAb levels were found in group 4 than in other groups.

Females predominated in all of the groups. However, a female sex prevalence was more visible in the AIT groups (**Table 1**).

Interestingly, in group 1, there were more cases with a positive family history of thyroid disorders (nodular goiter, AIT, and PTC) than in groups with PTC detected upon admission. In group 3, this percentage was only 13.3%, and it may explain why these patients presented with a more advanced disease stage upon the first referral (**Table 1**).

### 3.1 Admission

The causes of referral were as follows: in group 1, there were abnormal US features of the thyroid gland in over 70% of the patients, whereas goiter dominated in groups 2 to 4 (**Table 1**)

There were no significant differences in age upon admission between the groups (**Table 1**).

At AIT diagnosis, the mean thyroid volume was above the reference ranges for Polish patients and, according to WHO reference ranges, in all groups (32, 33). However, in groups 2 and 3, this was not statistically significantly bigger than in the other groups (**Table 1**).

There were significant differences between TSH, TPOAb, and TgAb between group 4 and the other groups as there were more cases of overt hypothyroidism in this group (**Table 1**).

In group 1, TSH ranged from 0-26, in group 2 from 0-6.2, in group 3 from 0.7-5.1, and in group 4 from 0-692.3 µIU/ml.

Hypothyroid patients from three groups started receiving levothyroxine: 22 patients in group 1 (4 with overt hypothyroidism and 18 with subclinical hypothyroidism), 4 patients in group 2 (all with subclinical hypothyroidism) and 60 patients in group 4 (55 with overt hypothyroidism and 5 with subclinical hypothyroidism).

Prior to surgery, three patients from group 2 were treated with the antithyroid drug methimazole due to thyreotoxicosis.

Interestingly, the TgAb/TPOAb ratio was significantly lower in group 4 [0.77 (1.9)] versus group 3 [3.5 (8.8)] and not significantly different versus group 2 [15.3 (27.6)] (**Table 1**).

### 3.2 PTC diagnosis

PTC was diagnosed at the mean time of 3.6 y (3 mo to 9 y) from AIT confirmation in group 1 (**Table 2**).

Significantly larger PTC diameters were found in groups 2 and 3 than in group 1 (**Table 2**). However, the localization of lesions (unifocal or multifocal and bilateral), number of lymph nodes with central or lateral PTC metastasis [lymph nodes metastasis (LNM)], maximum length of the largest metastatic lymph node, labelling index Ki67, extrathyroidal extension, and angioinvasion were similar in all groups 1-3 (**Table 2**).

After follow-up we have seen a decrease of mean TSH in group 1 and 4, however the mean thyroid volume decreased more in group 4 than in group 1. The mean (SD) TSH levels in group 1 and 2 were close to upper normal range and in the middle of the normal range in group 4.

TPOAb was significantly higher in group 4 than in the other groups. Interestingly, the TgAb/TPOAb ratio was significantly lower in group 4 [0.21 (0.4)] than in groups 3 [3.5 (8.8)] and 1 [5.2 (13.5)] (**Table 2**). This observation can be explained by a decrease of TPOAb level and increase in TgAb titers as seen in some children in group 1, whereas in the AIT group, we observed a decrease in both TPOAb and TgAb levels. Unfortunately, our small sample size enabled a more detailed analysis.

Histological assessment revealed more aggressive subtypes of PTC in group 2 and mostly in group 3: tall cells, diffuse sclerosing, and solid and more mixed subtypes, including the solid/anaplastic subtype (**Table 2**).

In group 1, only 71.4% were referred for 131I therapy. Meanwhile, this percentage was higher in group 2 (94.4%) and group 3 (93.3%) (**Table 2**).

### 3.3 US assessment

**Figure 1:**
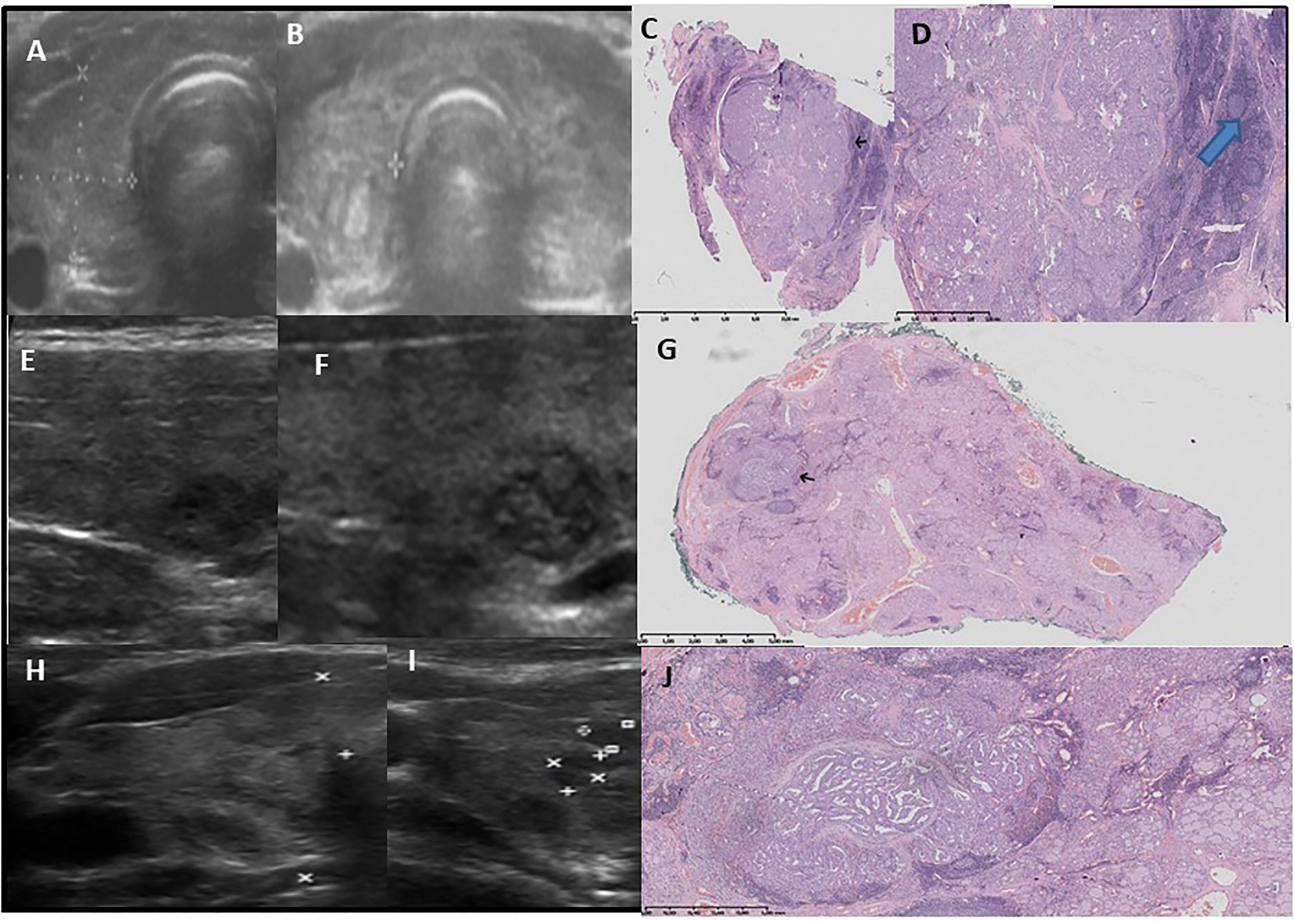
Pattern 1 - a lobulated nodule on US seen as few small malignant lesions- separated by fibrosis and surrounded by lymphocytic inflammation in histology. Ultrasound -pathology correlations. 1st row **(A–D)**: Development of lobulated nodule with nodular hyperechogenic solid composition after 5 years since the confirmation of autoimmune thyroiditis and first thyroid ultrasound. Increase in background echogenicity is visible. Female patient, age 17 years old, classic PTC, 10 mm, multifocal, bilateral **(A)**-before, **(B)**-after.**(C)** Lobulated tumour radiates from a central scar, pushing into thyroid H&E x1, **(D)** thin fibrotic capsule partially visible. Outside the tumour chronic lymphocytic infiltration with formation of germinal centers, fibrosis is visible H&E ×10, black arrow – tumour, blue arrow – germinal center in lymphocytic inflammation. 2nd and 3rd row **(E–J)**: Development of lobulated nodule with nodular hyperchogenic solid composition after 4 years since the confirmation of autoimmune thyroiditis and first thyroid ultrasound. Female patient, 18 years old, with classic, follicular PTC, 5 mm, multifocal, bilateral **(E, H)** -before, **(F, I)**-after). **(G)** Lobulated tumour with fibrotic layers inside, pushing into thyroid H&E x1, black arrow – tumour. **(J)** light-pink fibrotic capsule is partially visible in a small, central compartment with purely papillary composition. Tumour invades capsule and the outer part of whole cancer has more compact papillary-follicular composition surrounded by a dark-blue lymphocyte inflammation. The pink, thin, long fibrosis is present in non-tumoural thyroid. Angioinvasion (+). H&E ×20.

**Figure 2:**
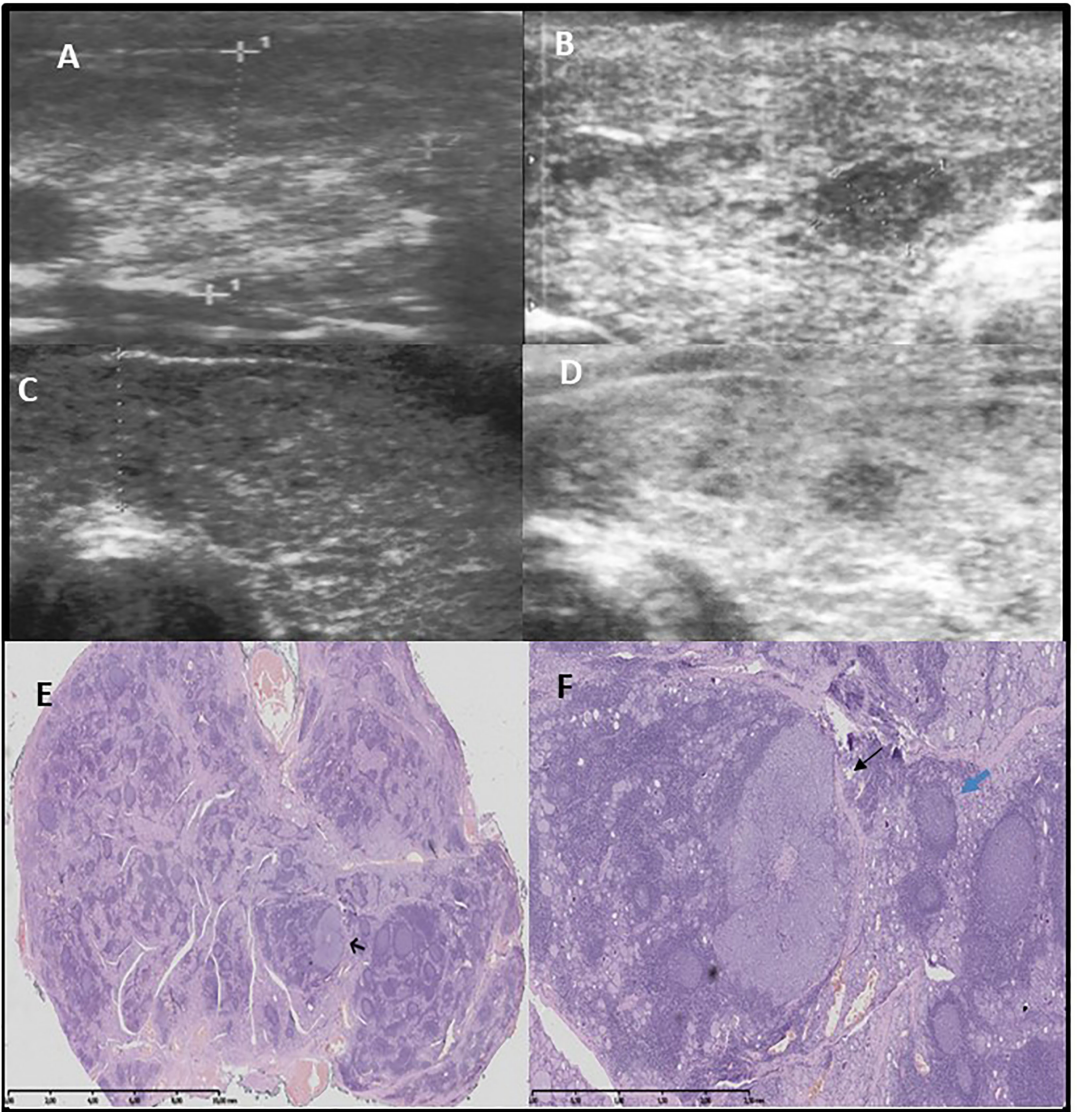
Pattern 3 - hypoechogenic nodule, histologically unencapsulated and with irregular margin. Row **(A, B)**: Development of hypoechogenic lesion surrounded by hyperechogenic fibrotic parenchyma after 1 9/12 years since the confirmation of autoimmune thyroiditis and first thyroid ultrasound. Female patient 11 years old, classic PTC, 6.7 mm, unilateral **(A)** before, **(B)** after Row **(C–F)**: Development of hypoechogenic lesion surrounded by hyperechogenic fibrotic parenchyma after 4 years since the confirmation of autoimmune thyroiditis and first thyroid ultrasound. Female patient 17.5 years old, classic, follicular PTC, multifocal, bilateral **(C)** before, **(D)** after, **(E)**The thyroid with AITD and goiter seen as a mixture of normal thyroid with colloid production, intense lymphocytic infiltration, as well as oblong and irregular fibrotic bands. In one large area of intense lymphocytic infiltration an irregular demarcated tumour is present (black arrow). H&E x1, **(F)**irregular classic PTC with lymphocytic background adhere on the right to fibrotic band (black arrow). The tumour is surrounded by large lymphocytic aggregation on the left. In the central part of the tumour, small, irregular, oval area of fibrosis is seen. H&E x10, blue arrow germinal center.

**Figure 3:**
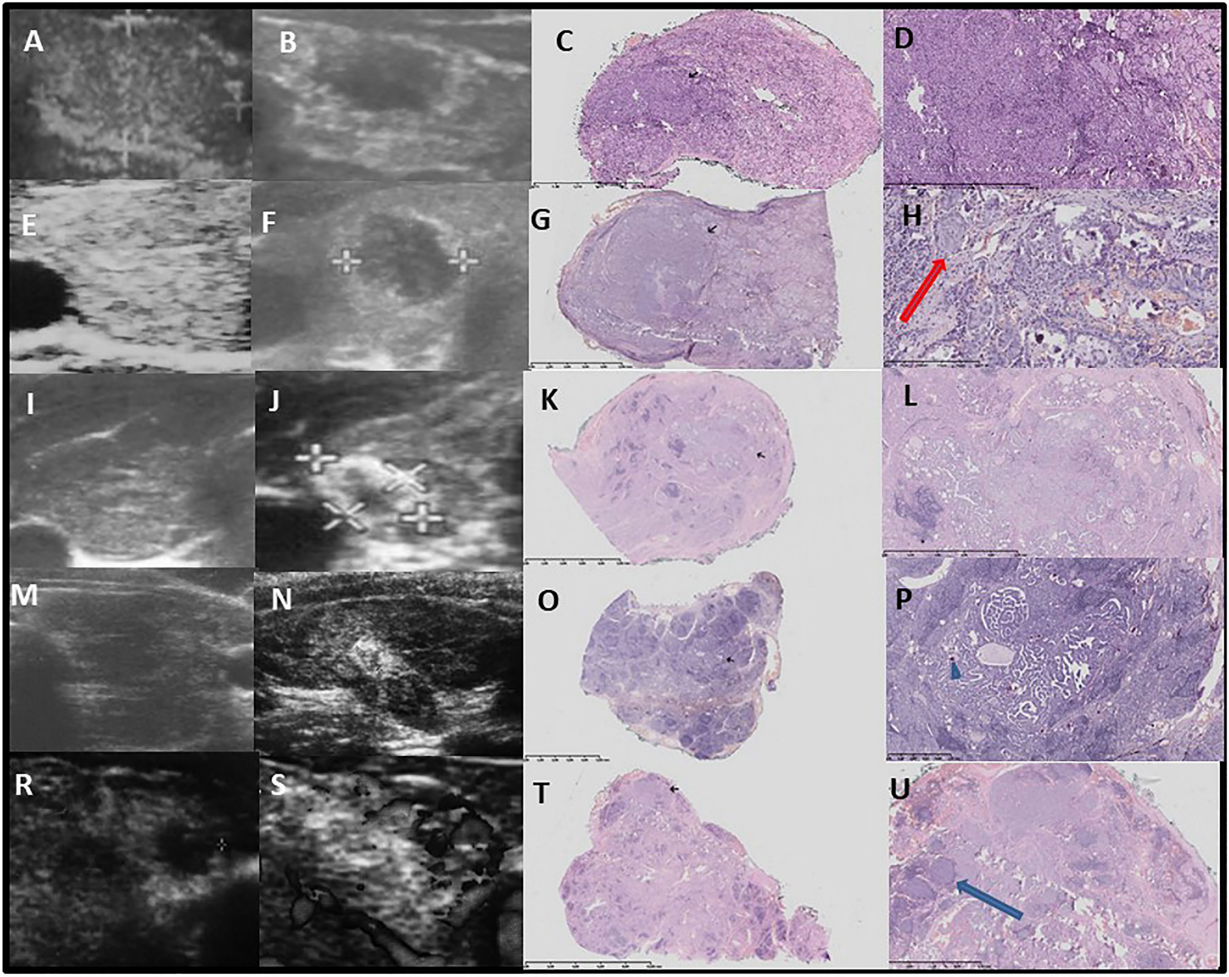
Pattern 2 – well circumscribed hypoechogenic lesion surrounded by a hyperechogenic thick layer and further, hypoechogenic parenchyma with hyperechogenic fibrosis. Thick layer is histologically seen as a tumour surrounded by fibrosis and psammoma bodies, and with diffuse thyroiditis in the background. Ultrasound -pathology correlations. Row **(A–D)**: Development of hypoechogenic nodule surrounded by hyperechogenic fibrotic layer after 4 7/12 years since the confirmation of autoimmune thyroiditis and first thyroid ultrasound. Female patient, 14 years old, classic, follicular, solid PTC, 7 mm and 2 mm, multifocal, bilateral **(A)**-before, **(B)**-after. **(C)** The tumour infiltrates into surrounding thyroid on histology, correlated with blurred margin on ultrasound, no angioinvasion H&E x1, black arrow – tumour. **(D)** thin fibrotic capsule around classic PTC subtype is visible (upper part of the tumour). Outside the tumour chronic lymphocytic infiltration (dark blue) and fibrosis with few psammoma bodies (small, purple bodies which generate oblong artefacts resembling jagged tissues) is visible H&E ×40. Row **(E–H)**: Development of hypoechogenic nodule surrounded by hyperechogenic fibrotic layer after 4 years since the confirmation of autoimmune thyroiditis and first thyroid ultrasound. Female patient, 13 years old, classic, follicular, solid PTC, 9 mm, unilateral **(E)** before, **(F)** after. **(G)** The ‘mushroom-shaped’ tumour, predominantly papillary in upper part, and follicular and solid in lower part which infiltrates into surrounding thyroid (histology) what correlates with blurred margin on ultrasound. Angioinvasion is present. Inside the solid part of the tumour oblong, irregular fibrosis and desmoplasia are visible. Tumour infiltrates its capsule (a thin capsule is seen in upper part of the tumour, which in this part is quite well demarcated and oval). H&E x1, black arrow – tumour. **(H)** High magnification of the PTC enriched with multiple psammoma bodies and a foci of squamous epithelial metaplasia (red arrow). H&E ×100. Row **(I–L)**: Development of hypoechogenic nodule surrounded by hyperechogenic fibrotic layer on the background of hypoechoic thyroid, after 4 11/12 years since the confirmation of autoimmune thyroiditis and first thyroid ultrasound. Female patient, 17.5 years old, classic, follicular, solid PTC, 9.8 mm, multifocal, bilateral **(I)** before, **(J)** after). **(K)** The tumour is infiltrating into surrounding thyroid on histology, angioinvasion is absent. H&E ×1, black arrow - tumour. **(L)** a biphasic (central – dense and solid, while outer – papillary and follicular) tumour infiltrates its capsule (herein a thin capsule demarcate upper part of the tumour). Outside the tumour chronic lymphocytic infiltration and irregular fibrosis is visible H&E ×10. Row **(M–P)**: Development of hypoechogenic nodule surrounded by hyperechogenic fibrotic layer on the background of hypoechoic thyroid, after 2 years since the confirmation of autoimmune thyroiditis and first thyroid ultrasound. Female patient, 8.8 years old, classic PTC, 6.7 mm, unilateral **(M)**-before, **(N)**-after). **(O)**-The tumour is not encapsulated and infiltrates the thyroid. Outside and inside the tumour massive lymphocytic infiltration with formation of germinal centres. H&E x1, black arrow – tumour. **(P)**-Papillary tumour with a few psammoma bodies (blue arrowhead) and fibrosis (surrounds the lower part and sides of the tumour as well as occurs between lymphocytic infiltration), H&E x40. Row **(R–U)**: Development of hypoechogenic nodule surrounded by hyperechogenic fibrotic layer after 4 years since the confirmation of autoimmune thyroiditis and first thyroid ultrasound. Female patient 17 years old, follicular PTC, 10 mm, multifocal, bilateral **(R)** before, **(S) **after). **(T) **Extensive fibrosis and lymphocytic infiltration of thyroid with less fibrosis in a tumour. H&E x1, black arrow – tumour. **(U) **Follicular tumour interspersed with thin bands of fibrous tissue. A few small psammoma bodies (which give oblong artefacts) are seen in lower part of the tumour. Lymphocytic germinal centers within the tumour (blue arrow) H&E x40.

US and corresponding histopathological features of the thyroid gland from representative patients from group 1 are presented in **Figures 1**–**3**.

Upon admission, in group 1, the US evaluation revealed a hypoechogenic thyroid gland in 12 and irregular normoechogenic gland in 16 patients. US monitoring showed an increase in thyroid echogenicity during the follow-up period in all of the patients in group 1; an increasing irregularity of the thyroid structure was also seen. Such changes were not noticed in the patients in group 4 with hypoechoic thyroid glands typical for diffuse thyroiditis during the study period. In groups 2 and 3, with PTC detected upon admission, the US of the thyroid gland represented nodular rebuilding in all of the patients at the background of irregular normoechogenicity of the thyroid gland.

The malignant lesion was hypoechoic in all of the patients, though we observed three US patterns. The first was seen in 18 out of 28 patients in group 1, 10 out of 18 patients in group 2, and all 45 in group 3; here, we noted the nodular, lobulated composition of the lesion (**Figure 1**). Histopathological assessment revealed small malignant lesions separated by fibrosis (**Figure 1**). The second pattern was seen in only seven patients only in group 1. The lesion was circumscribed by a hyperechogenic layer, which was histopathologically confirmed to be fibrosis with psammoma bodies (**Figure 3**). This pattern was characteristic in patients with diffuse thyroiditis and hypoechoic parenchyma with hyperechogenic layers of fibrosis seen in the background. The third pattern included hypoechogenic nodules later described by pathologists to be lesions with spiculated, irregular margins surrounded by fibrotic parenchyma with multiple areas of lymphocytic infiltration in patients with goiter. This pattern was seen in 3 out of 28 patients in group 1 and 8 out of 18 patients in group 2 (**Figure 2**).

We found that the majority of detected nodules showed a fast growth rate over time, with volumes that doubled or even tripled within 2–8 mo of observation before FNAB was performed. This pattern of growth has already been described in our previous report (25).

### 3.4 Pathology

After FNAB based on the Bethesda criteria was conducted, total thyroidectomy with lymph node dissection where appropriate was performed in all of the patients (31). The recurrences were observed only in group 3, in five patients who were subsequently subjected to lateral lymph node dissection.

There was no correlation between the PTC subtype and the pattern of a nodule seen on US, though our sample may have been too small to observe the differences.

A histopathological assessment revealed that the malignant lesions were not encapsulated. Fibrotic capsules, if partially visible, were infiltrated by the branches of the lesions. In all of the patients, the lymphocytic infiltration around the tumor was visible. In patients with a normoechoic background, the infiltration was less advanced in the surrounding thyroid tissue than in patients with a hypoechoic background, where massive inflammation with germinal centres infiltrated by tumors was visible (**Figures 1**–**3**).

### 3.5 Correlations

There were no correlations between TSH, TgAb, and the extent of the disease. The only determined associations involving the extent of the disease were with age and PTC nodule diameter. The patients’ ages correlated negatively with PTC mm (r: -0.3, p=0.01), LNM mm (r: -0.38, p=0.048), number of central and lateral LNM (r: -0.36; r: -0.32, p=0.01), extrathyroidal extension (ETE) (r: -0.37, p=0.01), and angioinvasion (AI) (r: -0.3, p=0.02). Negative correlations were found between thyroid peroxidase (TPO) and PTC mm (r: -0.29, p=0.011) and AI (r: -0.26, p=0.011). The diameter of PTC in mm correlated positively with LNM mm (r: 0.39, p=0.02), number of central and lateral LNM (r: 0.29; r: 0.36, p=0.01), LI Ki67 (r: 0.32, p=0.012), ETE (r: 0.36, p=0.01), and AI (r: 0.3, p=0.02).The TgAb/TPOAb ratio correlated with AI (r: 0.26, p=0.01).

## 4 Discussion

In the present follow-up study thyroid malignancies were detected early *via* US before they were clinically apparent. A surgical work-up of all of the patients confirmed malignancies, and there were no surgical complications and unnecessary surgeries. We found that the majority of detected nodules showed a fast growth rate over time, with volume that doubled or even tripled within 2–8 mo of observation before FNAB was performed. This pattern of growth has already been described in our previous report (25).

In the present study, we observed that paediatric PTC is fast-growing and aggressive as even with the regular monitoring and detection of small lesions after thyroidectomy, over 70% of children in group 1 were referred for 131I therapy due to multifocality and bilaterality and LNM, a known observation in paediatrics (2, 6, 13, 24). However, this percentage was over 90% in other groups; thus, active surveillance might reduce the need for 131I therapy. Additionally, the diameter of the nodules was significantly lower in the actively surveilled group. Interestingly, we observed more aggressive and mixed PTC subtypes in groups detected upon admission (referred with goiter or nodules already found during US assessment). Furthermore, the percentage of those with a family history of thyroid disorders was much smaller in group 2 and especially in group 3 than in group 1; therefore, the children in these groups were referred relatively late to endocrinologists. The poor awareness of thyroid diseases by parents without familiarity with thyroid problems could impact late referrals. This finding is not surprising, but it may underline the need for a thorough check-up of family thyroid history in paediatric patients, as well as thyroid US checks in cases of neck goiter even if this history is negative.

This study is an expansion of our previous work presenting the follow-up of patients with AIT who did not appear to have nodules on the first US check-up, which was performed during the first referral visit, but who later developed nodules, as seen during subsequent visits (25). The patterns observed and described in our previous work and presented to all members of our team enabled an increase in the detection of PTC in our departments. In the present study, the mean time to PTC detection was 3.6 y. These data are consistent with those of the Italian study by Rizzo et al., demonstrating that the lag time between AIT diagnosis and PTC detection was approximately 5 years (34). The increase in the number of thyroid nodules during follow-up, but not PTC, has also been reported by Radetti et al. in a large study of paediatric patients with AIT (13). This difference could be related to the small size bias in our group of selected patients.

The US evaluation of thyroid nodules detected during follow-up revealed three patterns of a nodule. To our knowledge, this is the first study describing different patterns of a developing nodule in paediatric patients. The first, which was more common, was of a lesion with a nodular, lobulated, solid composition. The second was of a lesion circumscribed by a hyperechogenic layer, which was histopathologically confirmed to be fibrosis with psammoma bodies. This pattern was characteristically seen in patients with diffuse thyroiditis and hypoechogenic parenchyma with hyperechogenic layers of fibrosis seen in the background. The third pattern included hypoechogenic nodules later described by pathologists as lesions with spiculated, irregular margins surrounded by fibrotic parenchyma in patients with goiter. These observations require much larger groups of paediatric patients in multicentre studies for confirmation and the determination of molecular associations.

A histopathological assessment revealed that malignant lesions were not encapsulated. Fibrotic capsules, if partially visible, were infiltrated by the branches of the lesions. This was in contrast to usually encapsulated PTC nodules in adults (24, 27). In all of the patients, the lymphocytic infiltration around the tumour was visible. However, in patients with a normoechoic background, it was less advanced in the surrounding thyroid tissue than in patients with a hypoechoic background, where massive lymphocytic infiltration, with the formation of germinal centres within the tumour, was visible.

Oppenheimer et al. found that the subset of patients with nodular AIT who had normal background parenchyma is a particularly interesting group to consider in further research and proposed that these patients may not carry a preexisting clinical diagnosis of AIT (22). Therefore, it was suggested by other research groups that an autoimmune reaction might be secondary to developing PTC (35–38). In a study by Paparodis et al., patients with less destructive AIT were described to have a higher risk for differentiated TC than were patients with a clear destructive AIT (21). However, in our study, we were able to notice the formation of a nodule on a hypoechoic background in several patients. As reported by Lee et al., a sonographic AIT diagnosis is made based on the hypoechogenicity and heterogenicity of the thyroid gland (7). Thyroidal parenchymal stiffness takes place in patients with AIT due to the lymphocytic infiltration, fibrosis, and follicular destruction in the thyroid gland during the disease process. This can be seen on US as increased parenchymal echogenicity with time (39, 40). We suggest that we should search for lesions that are circumscribed by a fibrotic layer in these paediatric patients.

Another point of interest in this study is the observation that although all hypothyroid patients in group 1 received levothyroxine treatment and presented decreased thyroid volumes and TSH levels, this therapy failed to provide any protection from nodule development. However, in the group 1 that developed AIT we have not seen similar decrease of mean thyroid volume as in AIT control group 4. Corrias et al. and Mussa et al. presented that progressive increase in nodule diameter under levothyroxine therapy is a factor which seems to be significantly associated with the risk of malignancy, an observation reported also by other researchers (13, 14, 25, 41).

After follow-up we have seen a decrease of mean TSH in group 1 and 4. The mean TSH levels in group 1 were close to upper normal range and in the middle of the normal range in group 4. Mussa et al. and Zirilli et al. presented that persistently elevated TSH levels play an independent role as predictors of the likelihood of TC, in children and in adults (41, 42). Interestingly Mussa et al. presented that serum TSH in the upper normal range was consistently associated wih thyroid cancer in children (41). It has to be emphasized that there are no current guidelines related to the level of TSH suppression in AIT treatment in children, as a possible TC prevention, and our study was not designed to provide them. Recent studies presented however, that TSH suppressive therapy for TC is associated with increased cardiovascular risk and has a negative impact on cognition in children (43–45), therefore in our AIT patients we aimed at TSH within the normal range.

An interesting observation was related to the TPOAb and TgAb assessments. TgAb is generally used as a prognostic marker of PTC only after total thyroidectomy, but its preoperative value in patients with PTC with concomitant AIT is still unclear (46). The role of thyroid antibodies in inducing malignant transformation of the thyroid tissue, regional lymph node invasion, and even long-term disease-free survival is controversial (47–51). Jia et al. and Kim et al. have reported for the first time that positive serum TgAb is an independent predictive marker for thyroid malignancy in patients diagnosed with thyroid nodules, regardless of the presence of AIT (52, 53). Interestingly, in our study, the TgAb/TPOAb ratio was significantly lower in children with AIT than in children in whom PTC was diagnosed upon admission, independently with regards to AIT. However, in patients with PTC who were not diagnosed with AIT, both TgAb and TPOAb levels were below the lower norm. Interestingly, in the group that was actively surveilled, the TgAb/TPOAb ratio increased since the beginning of the observation period and was higher than in patients with AIT. This observation can be explained by a decrease of TPOAb level and increase in TgAb titers as seen in some children in this group, whereas in the AIT group, we observed a decrease in both TPOAb and TgAb levels.

Recent studies by Hosseini et al. and Grani et al. have also suggested the use of preoperative TgAb levels as a marker for PTC (54, 55). The causal relationship between AIT and PTC is not yet clear, and it is uncertain whether TgAb is generated by the same pathological process in both AIT and PTC. It was found that the pattern of Tg recognition differs in patients with and without AITD (35, 56, 57). The Tg epitopic regions in patients without AITD were more variable than in those with AITD. Moreover, the pattern of recognition of TgAb in patients with both PTC and AIT was more similar to that observed in those with AIT, as compared to patients with PTC (35, 56, 57).

Min et al. observed an association between high serum TgAb levels and C LNM in patients with PTC and AIT, suggesting that it can be useful in predicting LNM in these patients (51). Similarly to our observation in paediatric patients that the TgAb/TPOAb ratio is associated with angioinvasion, Min et al. identified that the TgAb/TPOAb ratio were significantly associated with the extent of the disease once the ratio index was higher than 2 in adult patients (51). Unfortunately, a small sample size precluded a more detailed analysis in our study; therefore, multicentre paediatric research is necessary to confirm this observation.

Xu et al. have reported that larger PTC lesions and LNM were significantly associated with higher TgAb, and an increasing preoperative TgAb level of up to 2000 IU/mL was associated with shorter recurrence-free survival (46). According to Li et al., although preoperative positive TPOAb and TgAb are independent predictive markers for PTC, they are also associated with milder clinicopathological features of PTC (58). In our study, however, we found that AIT was observed in 50% of operated children, but we could not confirm the association of AIT with the PTC stage in relation to LNM or ETE. The only association involving the extent of the disease was found in relation to the age, tumour diameter, and TPOAb, similarly as observed in other recent paediatric studies (59–61). In paediatric patients, Huang et al. found that preoperative positive TgAb and TPOAb were protective factors for recurrence in younger age groups but not in older patients (62).

Unlike adults, children with differentiated TC may present with more advanced disease and have a higher local recurrence and distant metastases, even though their prognosis is favourable with overall 10-y survival rates of over 90% (24). Children have a longer posttreatment life expectancy and, thus, have more time for recurrence or potential treatment side-effects to manifest (1). Recent studies also confirmed that 131I therapy is associated with an increased risk for the development of second malignancies as well as with an increase in overall mortality for patients with PTC, especially for those who were treated in childhood (63). On the other hand, inadequate treatment of the initial cancer is linked to recurrent or persistent disease, and subsequent surgeries are much more difficult and prone to complications (1). Therefore, PTC prognostics is at present an area of intense research in the field of oncology (53).

Several limitations existed in our study. Due to the rarity of PTC incidence in young patients, our sample was small and had no power to establish statistical significance. Additionally, not all of the patients had repeated all of the tests, which influenced the result.

However, strengths included the fact that at the time of writing, the authors are clinical doctors and are involved in the diagnostic and therapeutic process. Additionally, patterns of nodule recognition described by our group are of practical value in every day US practices. Similarly to adult studies, we found that the preoperative TgAb/TPOAb ratio could likely serve as a novel prognostic factor for predicting PTC in children, though this needs confirmation in multicentre studies.

## 5 Conclusion

The use of thyroid US focused on the search for developing tumours in the routine follow-up of patients with AIT may not only help in the early detection of thyroid malignancies that are not clinically apparent but may also influence the invasiveness of oncological therapy and reduce the future side effects of 131I therapy. We propose that the repeat evaluation of TPOAb and TgAb warrants further exploration as a strategy to determine TC susceptibility in paediatric patients with AIT in larger multicentre studies.

## Data availability statement

The raw data supporting the conclusions of this article will be made available by the authors, without undue reservation.

## Ethics statement

This study was approved by the relevant institutional review board (The Bioethics Committee of the Jagiellonian University; opinion number:1072.6120.288.2021). Written informed consent to participate in this study was provided by the participants’ legal guardian/next of kin. Written informed consent was obtained from the minor(s)’ legal guardian/next of kin for the publication of any potentially identifiable images or data included in this article.

## Author contributions

Study design: DJ. Study conduct: DJ, AT-N, AK-W, MK, MW, WG. Data collection: DJ, MK, AT-N, AK-W, MW. Data analysis: DJ, MW, MK. Data interpretation: DJ, MW, MK, AT-N, AK-W. Drafting manuscript: DJ, MW. Revising manuscript content: DJ, MW, WG, JS. Approving final version of manuscript: DJ, MW, JS, WG. DJ takes responsibility for the integrity of the data analysis.
